# Assessing Psychological Fitness to Drive for Intoxicated Drivers: Relationships of Cognitive Abilities, Fluid Intelligence, and Personality Traits

**DOI:** 10.3389/fpsyg.2020.01002

**Published:** 2020-05-26

**Authors:** Martin Nechtelberger, Thomas Vlasak, Birgit Senft, Andrea Nechtelberger, Alfred Barth

**Affiliations:** ^1^Department of Social Sciences, University of Nicosia, Nicosia, Cyprus; ^2^Department of Psychology, Sigmund Freud University Linz, Linz, Austria; ^3^Department of Psychology, Austrian Academy of Psychology, Vienna, Austria

**Keywords:** traffic psychology, intoxicated drivers, psychological assessment, impaired driving, drunk driving, cognitive abilities, driving-related personality traits, fluid intelligence

## Abstract

Our study explores the relationships between traffic-psychological driving-related personality traits, fluid intelligence, and cognitive abilities for drivers whose driver license has been revoked due to intoxicated driving (alcohol and/or drugs). We were able to show that high significant impacts on cognitive functions derive from the participants’ age and fluid intelligence. In addition, driving-related personality traits like emotional instability, sense of responsibility and self-control contributed significantly to some of the cognitive abilities that are important for the fitness to drive. Additionally, mediating effects of fluid intelligence in the model are discussed. Traffic psychologists can use this knowledge in their assessment of drivers, mainly regarding the possible compensation of cognitive deficits regarding the fitness to drive.

## Introduction

About 25,600 people died and more than 1.4 million people were injured in car accidents in the Member States of the European Union in 2016 (European Union, 2018). In most countries worldwide, road traffic crashes cost around 3% of their gross domestic product (World Health Organization, 2018). These numbers show that the reduction of traffic accidents has to be a priority target for society in order to reduce emotional and physical pain as well as financial losses.

Drivers who were caught by the police with a level of 1.6 per thousand blood alcohol or higher or impairment because of the consumption of drugs have to prove their ability to drive in a psychological test if they want to get their revoked driving license back again. In addition, high fines are imposed and revocation periods have to be completed. The guidelines on how to conduct these tests are regulated in the Austrian Regulation on Health and Driving Licenses (Federal Republic of Austria, 2016). Risser et al. (2008) and Sommer et al. (2008b) have shown that standardized psychological driving tests are an appropriate criterion to measure the fitness to drive.

Since the act of driving requires a network of various cognitive abilities (Organisation for Economic Co-operation and Development, 2001), extensive research on the topic of driving ability and the identification of relevant cognitive domains has been carried out. A systematic review (Gard et al., 2014) showed that especially attention, executive functions, memory, perception, and coordination were the most important factors regarding driving a motor vehicle. These findings are in accordance with the results of a recent meta-analysis linking impairment in these specific cognitive domains to reduced driving performance (Hird et al., 2016). Finally, research has highlighted that age-related changes as well as the influence of fluid intelligence have to be taken into consideration when assessing these cognitive abilities (Hartshorne and Germine, 2015).

Sommer et al. (2008b) showed in the artificial neural network that, in addition to cognition, driving-related personality traits also indicate predictive relevance regarding fitness for driving. The study particularly connected sensation-seeking, social responsibility, self-control, and emotional instability to driving performance. The results of a recent study (Šucha and Černochová, 2016) emphasize the importance of personality by linking seeking for excitement, low self-control and a low sense of responsibility to reduced driving performance. Moreover, Classen et al. (2011) found out that extraversion may emphasize increased fitness to drive while its counterpart, introversion, may be linked to the opposite.

Although cognitive abilities and personality traits function as separate predictors for driving performance, the literature suggests a possible interdependence. A recent study by Sutin et al. (2019) found out that neuroticism is associated with worse performance in memory, psychomotor speed, attention and executive functions as well as visuospatial skills. On the other hand, consciousness, openness and agreeableness seem to have a positive influence on most of the cognitive domains, while extraversion may enhance speed, attention and executive function in particular (Sutin et al., 2019). Additionally, the results of Rammstedt et al. (2018) recently connected emotional stability to higher performance in cognitive tasks, whereas Colom et al. (2007) found that high levels of sensation-seeking as well as impulsiveness, which are parts of the trait of self-control, are linked to slower perceptual speed. Moreover, social responsibility was also found to play a supporting role in maintaining cognitive performance (Secchi, 2009). Finally, various studies note possible interactions between personality and fluid intelligence (e.g., Baker and Bichsel, 2006; Zimprich et al., 2009).

Compensating for the test results of participants who fail these conditions in some categories is possible on an individual basis. There is some general advice on rules for such compensation (Schubert et al., 2018), but it only provides the traffic psychologist with rough outlines. Since there is hardly any research in this field, the objective of our study is to provide an initial understanding of the interdependences between cognitive tasks, driving-related personality traits, and fluid intelligence regarding fitness to drive. In addition, possible mediators are identified in terms of potential compensating effects of cognitive deficits to provide a deeper understanding for traffic psychologists. Therefore, recommendations on the compensation of participants’ test results are given in order to generate a deeper understanding for traffic psychologists regarding the mentioned uncertain outlines.

### Structure of the Traffic Psychological Assessment in Austria

The cognitive abilities which have to be checked for the specific skill of driving a motor vehicle are obtaining an overview in traffic, ability to react, speed of reactions and stress tolerance in reactions, concentration, eye-hand coordination, and memory. Logical reasoning is assessed as a measure for fluid intelligence. The main personality dimensions regulated in Austrian law regarding the personal attitudes of drivers (personality traits) are a sense of responsibility, self-control, mental instability and readiness to take risks in traffic.

A traffic psychological report is written by the traffic psychologist and subsequently sent to the driving license authority. This report is based on the interview data, the psychological test data and behavioral observation. The result of this report is a recommendation to the driving license authority to reissue the driving license to the driver (with or without limitations) or to extend the period of revocation of the driving license.

Impaired drivers who apply for a general driving license (normal cars) have to prove that they have at least “average” test results (Caloupka-Risser et al., 2011; Schubert et al., 2018). The definition of “average” means that the percentile rank of every test result is larger than 15. DUI drivers applying for an occupational driving license (buses, coaches, and trucks) must prove that they meet the minimum requirements of a percentile rank of every test result of 33. The percentile rank is computed in relation to the norm of the general population.

This can be compensated for in the assessment by consideration of the participant’s mental status. Aspects which are included here are favorable personality aspects that are evident in the interview with the traffic psychologists and the results of the personality assessment. A better knowledge of the relationship between cognitive abilities, fluid intelligence and personality traits for intoxicated drivers would thus be very important in this field. Deeper insights will help traffic psychologists to be able to assess possible compensatory factors for deficits in the fitness to drive assessment more accurately in the future.

## Materials and Methods

### Questionnaires

The traffic psychology assessment in Austria uses the following psychological questionnaires and tests. We will describe them briefly and introduce the main variables that our study addresses.

#### Hand-Eye-Coordination Speed and Quality (2HAND)

The 2HAND test focuses on sensorimotor coordination between eye and hand and coordination between the left and right hands. A red dot has to be moved along a given track. The up and down direction is controlled with one hand and the left and right coordination is controlled with the other hand. All details regarding the test can be found in Puhr (2011). The subform S4 (10 runs, long version) has been used. The main variable is the total mean duration as a measure of speed of movement and thus the level of performance and the total percentage error duration as a measure of the quality of performance. Higher scores indicate a lower level of fitness to drive. Validity is not only provided by its logic but also by a significant correlation between the test and the assessment of driving ability regarding criterion validity (*r* = 0.50, *p* < 0.001). In addition, construct validity is given by significant intercorrelations between test variables (*r* = 0.32, *p* < 0.001; *r* = 0.84, *p* < 0.001) (Karner and Neuwirth, 2000). In our sample an internal consistency of α = 0.87 was given for hand-eye-coordination quality and a Cronbachs Alpha of 0.91 for hand-eye-coordination speed.

#### Selective Attention (COG)

The test assesses attention and concentration through the comparison of figures with regard to their congruence. A geometric figure is compared with four other geometric figures. It then has to be indicated whether the comparative figure is identical to one of the other figures (Wagner and Karner, 2012). The subform used was S11 (short form with free working time). The main variable is mean time “correct rejection”. Higher scores represent a lower level of fitness to drive. Construct validity is given by significantly positively correlating the test form to the model of concentration (Reulecke, 1991; Wagner, 1999), whereas criterion validity was supported by many studies regarding convergent and divergent correlations (e.g., Karner, 2000; Neuwirth, 2001; Sommer and Häusler, 2006). Internal consistency was estimated of α = 0.93 in our sample.

#### Resilience of Attention (DT)

The DT test is used to measure reactive stress tolerance and the associated ability to react. Different optical and acoustic stimuli have to be responded to by pressing corresponding keys. We used the subform S5 (Vienna form A). The main variable is the median reaction time. Higher scores indicate a lower level of fitness to drive. Extreme-group validity for the test is supported by the study of Karner (2000) by significantly distinguishing between the norm population and the alcohol-related offense group, while Karner and Neuwirth (2000) found significant positive correlations between the results of DT and driving test. In addition, significant intercorrelations regarding construct validity were found ranging from *r* = 0.90 to 0.40. A Cronbach’s alpha of 0.96 was estimated for the included sample.

#### Driving-Related Personality Traits (IVPE)

The test is described by Sommer et al. (2011). It is used to measure personality traits which are relevant for driving: Sense of Responsibility, Self-Control, Adventurousness and Need for Excitement and Emotional Instability. Adventurousness and Need for Excitement is a driving-related personality trait that is based on the construct of sensation-seeking (Zuckerman, 1994). Higher scores in Emotional Stability, Adventurousness and Need for Excitement indicate lower levels of fitness to drive, whereas lower scores in Sense of Social Responsibility and Self-Control also represent lower levels of fitness to drive. It is worth pointing out that the scale of Emotional Instability in the questionnaire is in reversed pooling. Construct validity is given by the high correlation of Emotional Instability and the subscale neuroticism from the big five personality model (McCrae and Costa, 1997; Rothmann and Coetzer, 2003). Furthermore, the scale Adventurousness and Need for Excitement highly loads on the subscale of sensation seeking of the Eysenck Personality Profiler (Eysenck et al., 2000). In addition, the trait Self-Control is based on the general theory of crime (Gottfredson and Hirschi, 1990) while Sense of Social Responsibility is based on the theory of pro-social behavior (Bacher, 2000) and the three-component model of Stahlberg and Frey (1996). Construct validity was supported by confirmatory factor analysis and following the exact model fit extreme-group validity was also given (Sommer et al., 2004). The reliability of each driving-related personality trait is given due to the fit of the Rasch model (Rasch, 1980). Since Cronbach’s alpha ranged from α = 0.82 to 0.85 in our sample, internal consistency was given for all the difference personality scales.

#### Reaction Speed and Physical Motor Speed (RT)

The participants have to press or release a button as quickly as possible when a light stimulus, a sound stimulus or a combination of these two stimuli is given. The subform S3 was used. Participants have to react when the simultaneous event of yellow light and sound occurs. The main variables are mean reaction time and mean motor time. Higher scores reflect a lower level of fitness to drive. Construct validity was not only supported by significant intercorrelations in the subform used ranging from *r* = 0.40 to 0.26 (both *p* < 0.001) but also by convergent validation with the Vienna Test System, while *r* = 0.68 was given for reaction speed and *r* = 0.68 for physical motor speed (Karner and Biehl, 2001). Regarding reliability, a Cronbach’s alpha of α = 0.94 for reaction speed and α = 0.97 for physical motor speed were estimated for our sample.

#### Fluid Intelligence (SPM)

An abstract picture is shown to the participant while one section of the image is missing. A series of suggested sections are given to the test person in order to complete the picture. Since the image follows a certain pattern, the participant has to figure out which section is the most appropriate for completion of the picture. The test measures fluid intelligence, which describes problem solving capacities based on logical reasoning and without any previous knowledge (Jaeggi et al., 2008). The form S5 (15 min’ time limit) was used. The main variable is the total of correct answers. Lower scores indicate a lower level of fitness to drive. Concerning validity, the SPM correlates with other intelligence tests in a range of *r* = 0.54–0.86, while factor analysis showed high loads up to *r* = 0.94 to the g-factor. Internal consistency is given ranging from α = 0.77 to 0.99 while split half-reliabilities range up to *r* = 0.90 (Raven et al., 2000). We were able to estimate a Cronbach’s alpha of 0.88 in our included sample.

#### Perceptual Speed (TAVT)

The TAVT is a test regarding the performance and the speed of comprehension through the brief presentation of images showing traffic situations. Before every picture acoustic stimuli are given to the participant. After every image the test person has to choose which element was shown from five possible answers. The test is fair, since neither traffic experience nor a knowledge of rules are an advantage. The subtest version S1 was used. The main variable is the level of overview, which is the number of traffic situations where the subject answered completely correctly. Lower scores in perceptual speed represent a lower level of fitness to drive. Construct validity is supported by the study of Sommer et al. (2008a) as well as content validity regarding research of extreme group validity (Sommer et al., 2005) and convergent validity (Risser et al., 2008). Internal consistency follows the Rasch model (Rasch, 1980). In terms of internal consistency, a Cronbach’s alpha of 0.84 could be estimated for our sample.

#### Short-Term Memory (VISGED)

This test is described in the manual by Hornke et al. (2011). The test assesses visual memory performance by measuring how respondents receive and reproduce visual information given by the positions of symbols on a city map. The main variable is visual memory performance. Lower scores in short-term memory indicate a lower level of fitness to drive. The test follows logical validity as well as construct validity. Estimating internal consistency, a Cronbach’s alpha of 0.84 was given regarding our sample.

### Participants

Since 2004 when the study was initially planned, field data has been collected from the computerized Schuhfried test systems in our traffic psychology institute in the greater Vienna region. Participants were recruited by the Traffic Psychology Examination Board (AAP), recognized by the Ministry of Transport, in the course of these measures to reissue driving licenses laid down under the Health Regulation of the Driving Licenses Act from 2004 to 2019. The traffic psychology assessment consisted of an extensive interview of the driver by a traffic psychologist as well as a clinical psychologist plus traffic psychology tests performed using the computerized Schuhfried test system. Participants who screened positively for any mental disorder, substance abuse, etc., in the interviews were not included in this study. The traffic psychologists asked the participants whether their data could be used for scientific purposes after explaining the aims and scopes of the study and guaranteeing the anonymity and confidentiality of the data. The participants were also assured that they would be able to withdraw without any negative consequences. Data was included in the study only in cases where the participants agreed to it. Neither agreement nor refusal to participate in the study had any impact on the traffic psychology assessment of their fitness to drive. The total sample of DUI drivers with complete predictor data sets for our study comprised *N* = 1,885 participants, of whom *N* = 230 (12.2%) were female and *N* = 1,655 (87.8%) were male. The minimum age was 18 years and the maximum age was 94.2 years with a mean of 39.68 years and a standard deviation of 12.93. The median is 38.7 years. The sample size varies for each regression analysis due to the data availability of each criterion. For a detailed description of the descriptive statistics regarding the applied measurements see Table 1.

**TABLE 1:** Descriptive Statistics.

Variable	Mean	Standard deviation	Median	Minimum	Maximum
Hand-eye-coordination speed (2HAND)	39.68	15.43	36.95	11.80	131.13
Hand-eye-coordination quality (2HAND)	3.87	3.83	2.69	0	26.80
Selective attention (COG)	2.82	0.85	2.64	1.20	10.10
Resilience of attention (DT)	0.78	0.12	0.76	0.50	1.40
Reaction speed (RT)	436.33	83.05	427.00	248.00	1044.00
Physical motor speed (RT)	153.93	56.25	144.00	39.00	579.00
Perceptual speed (TAVT)	11.75	3.14	12.00	0.00	20.00
Short term memory (VISGED)	1.77	1.10	1.80	0.00	5.00
Sense of responsibility (IVPE)	7.49	2.37	8.00	0.00	10.00
Self-control (IVPE)	5.74	1.46	6.00	0.00	7.00
Adventurousness and need for excitement (IVPE)	4.40	2.40	4.00	0.00	10.00
Emotional instability (IVPE)	1.47	1.70	1.00	0.00	10.00

## Results

The hierarchical multiple regression analysis as well as mediating effects were analyzed in SPSS 25.0 (IBM Corp, 2017). We carried out hierarchical multiple regression models controlling for age and gender in the first step, defining a sense of responsibility, self-control, adventurousness and need for excitement and emotional instability as independent variables in the second step, adding fluid intelligence in the third and including the interaction terms of age and the driving-related personality traits of our analysis. Regression models were formed separately for each of the different cognitive abilities. A detailed overview regarding intercorrelations between study variables is given in Table 2 and summarized results of hierarchical regression analyses is presented in Table 3. Mediation analyses were carried out in SPSS 25.0 with the PROCESS 3.4 macro (Hayes, 2018). In this regard, we analyzed the direct and indirect effects of the driving-related personality traits on the different cognitive abilities when fluid intelligence was included as a mediator variable in the model. Significance of moderations was tested estimating indirect effects via 95% bias-corrected confidence intervals calculated through 10 000 bootstrap samples. Null hypothesis regarding mediation was rejected when the confidence interval did not include zero (Hayes, 2018).

**TABLE 2:** Correlations between study variables.

	Hand-eye-coordi- nation speed	Hand-eye-coordi- nation quality	Short term memory	Resilience of attention	Selective attention	Reaction speed	Physical motor speed	Perceptual speed	Advent- urous- ness and need for excitement	Emotional instability	Self control	Sense of respon- sibility	Fluid intelli- gence	Age	Sex
Hand-eye-coordination speed	1	−0.19**	−0.25**	0.45**	0.41**	0.26**	0.29**	−0.28**	−0.15**	0.08**	0.00	0.04	−0.28**	0.38**	0.11**
Hand-eye-coordination quality		1	−0.15**	0.19**	0.10**	0.09**	0.13**	−0.18**	–0.00	0.09**	−0.06*	−0.07**	−0.19**	0.10**	0.10**
Short term memory			1	−0.44**	−0.36**	−0.21**	−0.27**	.40**	0.10*	−0.14**	0.10*	0.03	0.43**	−0.38**	0.00
Resilience of attention				1	0.57**	0.48**	0.46**	−0.53**	−0.20**	0.16**	–0.02	0.06*	−0.46**	0.63**	0.12**
Selective attention					1	0.34**	0.31**	−0.40**	−0.18**	0.09**	0.00	0.09**	−0.42**	0.44**	0.01
Reaction speed						1	0.42**	−0.26**	−0.09**	0.06*	−0.06**	–0.03	−0.27**	0.31**	0.07**
Physical motor speed							1	−0.32**	−0.16**	0.12**	−0.06*	0.02	−0.30**	0.43**	0.18**
Perceptual speed								1	0.20**	−0.20**	0.04	–0.02	0.46**	−0.52**	−0.12**
Adventurousness and need for excitement									1	0.03	−0.34**	−0.33**	0.11**	−0.35**	−0.16**
Emotional instability										1	−0.09**	0.00	−0.16**	0.07**	0.07**
Self control											1	0.62**	0.05*	0.04	0.00
Sense of responsibility												1	0.00	0.16**	0.00
Fluid intelligence													1	−0.38**	−0.05*
Age														1	0.09**
Sex															1

*p < 0.05; **p < 0.001.

**TABLE 3:** Results hierarchical regression.

Summary hierarchical multiple regression on cognitive abilities								
	Hand-eye-coordi nation speed	Hand-eye-coordi nation quality	Selective attention	Resilience of attention	Reaction speed	Physical motor speed	Perceptual speed	Short-term memory
**MODELS**								
**Step 1: Demographics**								

Sex	*B* = 3.74, se = 1.14, β = 0.08**, *t* = 3.23	*B* = 1.10, se = 0.31, β = 0.10***, *t* = 3.60	*B* = −0.07, se = 0.05, β = −0.03, *t* = −1.32	*B* = 0.03, se = 0.01, β = 0.07***, *t* = 3.72	*B* = 10.85, se = 5.59, β = 0.04, *t* = 1.94	*B* = 24.41, se = 3.55, β = 0.14***, *t* = 6.89	*B* = −0.76, se = 0.19, β = −0.08***, *t* = −4.03	*B* = −0.02, se = 0.23, β = −0.00, *t* = −0.10
Age	*B* = 0.46, se = 0.03, β = 0.37***, *t* = 14.70	*B* = 0.03, se = 0.31, β = 0.09**, *t* = 3.34	*B* = −0.03, se = 0.00, β = 0.044***, *t* = 21.24	*B* = 0.01, se = 0.00, β = 0.62***, *t* = 34.15	*B* = 1.96, se = 0.14, β = 0.30***, *t* = 13.81	*B* = 1.84, se = 0.09, β = 0.42***, *t* = 20.43	*B* = −0.13, se = 0.01, β = −0.51***, *t* = −26.00	*B* = −0.06, se = 0.01, β = −0.38***, *t* = −10.21

	*R* = 0.385, *R*^2^ = 0.149, *R*^2^_*adj*_. = 0.147	*R* = 0.137, *R*^2^ = 0.019, *R*^2^adj. = 0.017	*R* = 0.443, *R*^2^ = 0.196, *R*^2^adj. = 0.195	*R* = 0.631, *R*^2^ = 0.398, *R*^2^adj. = 0.397	*R* = 0.311, *R*^2^ = 0.097, *R*^2^adj. = 0.096	*R* = 0.456, *R*^2^ = 0.208, *R*^2^adj. = 0.207	*R* = 0.525, *R*^2^ = 0.276, *R*^2^adj. = 0.275	*R* = 0.382, *R*^2^ = 0.146, *R*^2^adj. = 0.143

**Step 2: Personality**								

Emotional instability	*B* = 0.42, se = 0.23, β = 0.05, *t* = 1.83	*B* = 0.18, se = 0.06, β = 0.08**, *t* = 2.86	*B* = 0.03. se = 0.01, β = 0.07**, *t* = 3.10	*B* = 0.01, se = 0.00, β = 0.11***, *t* = 6.04	*B* = 1.60, se = 1.08, β = 0.03, *t* = 1.47	*B* = 2.69, se = 0.69, β = 0.08***, *t* = 3.92	*B* = −0.29, se = 0.04, β = −0.16***, *t* = −8.09	*B* = −0.10, se = 0.05, β = −0.08*, *t* = −2.11
Sense of responsibility	*B* = −0.11, se = 0.22, β = −0.02, *t* = −0.53	*B* = −0.14, se = 0.06, β = −0.08*, *t* = −2.40	*B* = 0.00, se = 0.01, β = 0.03, *t* = 0.97	*B* = −0.00, se = 0.00, β = −0.05, *t* = −1.92	*B* = −1.83, se = 1.00, β = −0.05, *t* = −1.83	*B* = −0.50, se = 0.63, β = −0.02, *t* = −0.78	*B* = 0.09, se = 0.03, β = 0.06*, *t* = 2.54	*B* = 0.04, se = 0.04, β = 0.04, *t* = 0.85
Self-control	*B* = −0.00, se = 0.35, β = 0.00, *t* = −0.01	*B* = 0.02, se = 0.09, β = 0.00, *t* = 0.17	*B* = −0.02, se = 0.02, β = −0.03, *t* = −1.18	*B* = 0.00, se = 0.00, β = −0.00, *t* = −0.13	*B* = −2.05, se = 1.63, β = −0.04, *t* = −1.25	*B* = −2.20, se = 1.03, β = −0.06*, *t* = −2.13	*B* = 0.04, se = 0.05, β = 0.02, *t* = 0.69	*B* = 0.13, se = 0.07, β = 0.09, *t* = 1.84
Adventurousness and need for excitement	*B* = −0.21, se = 0.19, β = −0.03, *t* = −1.10	*B* = 0.03, se = 0.05, β = 0.02, *t* = 0.53	*B* = −0.01, se = 0.00, β = −0.03, *t* = 0.15	*B* = 0.00, se = 0.00, β = 0.01, *t* = 0.64	*B* = 0.10, se = 0.88, β = 0.00, *t* = 0.91	*B* = −0.57, se = 0.56, β = −0.02, *t* = −1.02	*B* = 0.05, se = 0.03, β = 0.04, *t* = 1.81	*B* = 0.01, se = 0.04, β = 0.01, *t* = 0.24

	*R* = 0.389, *R*^2^ = 0.151, *R*^2^_*adj*_. = 0.148	*R* = 0.18, *R*^2^ = 0.032, *R*^2^_*adj*_. = 0.028	*R* = 0.449, *R*^2^ = 0.202, *R*^2^_*adj*_. = 0.199	*R* = 0.642, *R*^2^ = 0.413, *R*^2^_*adj*_. = 0.411	*R* = 0.323, *R*^2^ = 0.104, *R*^2^_*adj*_. = 0.102	*R* = 0.469, *R*^2^ = 0.220, *R*^2^adj. = 0.217	*R* = 0.553, *R*^2^ = 0.306, *R*^2^ adj. = 0.303	*R* = 0.408, *R*^2^ = 0.167, *R*^2^adj. = 0.158

**Step 3: Intelligence**								

Fluid intelligence	*B* = −0.45, se = 0.75, β = −0.16***, *t* = −6.03	*B* = −0.11, se = 0.02, β = −0.16***, *t* = −5.39	*B* = −0.04, se = 0.00, β = −0.29***, *t* = −13.41	*B* = −0.01, se = 0.00, β = −0.26***, *t* = −13.92	*B* = −2.51, se = 0.35, β = −0.17, *t* = −7.30	*B* = −1.45, se = 0.22, β = −0.15***, *t* = −6.62	*B* = 0.16, se = 0.01, β = 0.28***, *t* = 14.15	*B* = 0.16, se = 0.01, β = 0.32***, *t* = 8.02
	*R* = 0.417, *R*^2^ = 0.174, *R*^2^_*adj*_. = 0.170	*R* = 0.23 *R*^2^ = 0.053, *R*^2^_*adj*_. = 0.048	*R* = 0.522, *R*^2^ = 0.273, *R*^2^_*adj*_. = 0.270	*R* = 0.685, *R*^2^ = 0.469, *R*^2^_*adj*_. = 0.467	*R* = 0.359, *R*^2^ = 0.129, *R*^2^_*adj*_. = 0.126	*R* = 0.488, *R*^2^ = 0.238, *R*^2^adj. = 0.235	*R* = 0.610, *R*^2^ = 0.373, *R*^2^adj. = 0.370	*R* = 0.497, *R*^2^ = 0.247, *R*^2^adj. = 0.238

*p < 0.05; **p < 0.01; ***p < 0.001.

Multicollinearity was assessed for every regression model in terms of the values of variance inflation factors (VIF). No collinearity problems can be assumed due to the VIF values of the predictors ranging beneath the recommended threshold of 5 (Hair et al., 2010). In addition, no suppressor variables could be found in the analysis.

### Hand-Eye-Coordination Speed (2HAND)

The first regression model regarding the dependent variable handy-eye-coordination speed showed significant ANOVA results [*F*(2, 1,339) = 116.81, *p* < 0.001] explaining 14.90% of variance. Age (β = 0.37, *p* < 0.001) as well as gender (β = 0.08, *p* < 0.001) were significant predictors. When including driving-related personality traits, an increase of R^2^ to 15.10% was given, which was statistically non-significant in terms of change [*F*(4, 1,335) = 1.12, *p* = 0.334], therefore driving-related personality traits were non-significant predictors in the model. However, significant changes in explained variance up to 17.40% can be shown [*F*(1, 1,334) = 36.37, *p* < 0.001] when adding fluid intelligence (β = −0.16, *p* < 0.001) to the model [*F*(7, 1,334) = 40.13, *p* < 0.001]. Since fluid intelligence represents a significant negative predictor, it reduces the hand-eye-coordination speed for every point by 0.45, indicating a higher level of fitness to drive.

No significant mediating effects through fluid intelligence could be found regarding the driving-related personality traits.

### Hand-Eye-Coordination Quality (2HAND)

The first controlling regression model for hand-eye-coordination quality again resulted in significant model results [*F*(2, 1,339) = 12.82, *p* < 0.001] following an R^2^ = 1.90%. Age (β = 0.09, *p* < 0.001] as well as gender (β = 0.10, *p* < 0.001) are significant predictors. Regarding the second step – adding driving-related personality traits – there was a significant change in explained variance to 2.80% [*F*(4, 1,335) = 4.63, *p* = 0.001]. Emotional instability (β = 0.08, *p* = 0.004) as well as a sense of responsibility (β = −0.08, *p* = 0.017) can be shown as significant predictors in the model [*F*(6, 1,335) = 7.41, *p* < 0.001]. While emotional instability is a positive predictor increasing the dependent variable for every point by 0.18, indicating a lower level of fitness to drive, a sense of responsibility decreases hand-eye-coordination quality by 0.60, representing a higher level of fitness to drive. When including fluid intelligence in the last step of the analysis, a significant increase in R^2^ to 4.80% can be seen [*F*(1, 1334) = 29.03, *p* < 0.001]. Emotional instability (*p* = 0.036) and sense of responsibility (*p* = 0.040) remained significant, whereas fluid intelligence (β = −0.16, *p* < 0.001) is an additional significant predictor in the model [*F*(7, 1,334) = 10.63, *p* < 0.001]. Fluid intelligence as negative predictor reduces the dependent variable for every point by 0.11, representing a higher level of fitness to drive.

Regarding mediation analysis via fluid intelligence, a significant total effect of emotional instability on hand-eye-coordination quality could be found (β = 0.09, *p* = 0.005). In detail, the results showed an insignificant direct effect of emotional instability on the cognitive domain (β = 0.07, *p* = 0.16) while the indirect effect through fluid intelligence was found to be significant [β = 0.03, 95% (0.02–0.04)]. Therefore, potential full mediating effects by fluid intelligence regarding the relation between emotional instability and hand-eye-coordination quality can be stated. However, no further significant mediating effects via fluid intelligence could be analyzed regarding the remaining driving-related personality traits.

### Selective Attention (COG)

The first controlling regression model is significant [*F*(2, 1,852) = 225.78, *p* < 0.001] explaining 19.60% of the variance. Only age (β = 0.44, *p* < 0.001) was found to be a significant predictor. Regarding the second step including driving-related personality traits there was a significant increase in R^2^ to 20.20% [*F*(4, 1,848) = 3.31, *p* = 0.010], while emotional instability is a significant predictor (β = 0.07, *p* = 0.002) in the model [*F*(6, 1,848) = 77.84, *p* < 0.001]. When fluid intelligence is added to the model there is a significant change increasing explained variance to 52.20% [*F*(1, 1,847) = 179.78, *p* < 0.001]. Although emotional instability did not remain significant (*p* = 0.186) fluid intelligence (β = −0.29, *p* < 0.001) was found to be a significant predictor in the model [*F*(7, 1,847) = 98.86, *p* < 0.001]. Since fluid intelligence functions as negative predictor it reduces selective attention for every point by 0.04, reflecting a higher level of fitness to drive.

Considering fluid intelligence as a mediating variable, a significant total effect of emotional instability on selective attention was estimated (β = 0.09, *p* < 0.001). Furthermore, the direct effect of emotional stability on selective attention was found to be insignificant (β = 0.03, *p* = 0.21) while the indirect effect via fluid intelligence showed significance [β = 0.07, 95% (0.04–0.08)]. Henceforth potential full mediation of the relationship between emotional stability and selective attention through fluid intelligence can be noted. Again, no additional significant mediating effects could be found regarding the other driving-related personality traits.

### Resilience of Attention (DT)

In the first model significant results of the ANOVA can be shown [*F*(2, 1,837) = 606.52, *p* < 0.001] explaining 40.00% of variance. Age (β = 0.62, *p* < 0.001) and gender (β = 0.07, *p* < 0.001) are significant predictors. When including driving-related personality traits in the second step a significant change in R^2^ to 41.30% was noted [*F*(4, 1,833) = 11.55, *p* < 0.001]. Only emotional instability (β = 0.11, *p* < 0.001) could be found to be an additional significant predictor in the second model [*F*(6, 1,833) = 214.52, *p* < 0.001]. Since emotional instability serves as a positive predictor it increases the dependent variable for every point by 0.01, indicating a lower level of fitness to drive. When adding fluid intelligence to the regression, an increase in explained variance to 47.00% resulted [*F*(1, 1,832) = 193.70, *p* < 0.001]. Emotional instability remained significant (*p* < 0.001), and fluid intelligence (β = −0.26, *p* < 0.001) was found to be a significant predictor in the model [*F*(7, 1,832) = 230.88, *p* < 0.001]. Fluid intelligence therefore reduces the dependent variable for every point by 0.01 reflecting a higher level of fitness to drive.

Regarding the relation between emotional stability and resilience of attention, when fluid intelligence was included as a mediating variable, a significant total effect could be found (β = 0.16, *p* < 0.001). Although the direct effect of emotional instability remained significant (β = 0.09, *p* < 0.001) there was an additional significant direct effect through intelligence on resilience of attention (β = 0.07, 95% (0.05–0.09). Therefore, potential partial mediation of the relationship between emotional instability and resilience of attention can be assumed. However, no significant mediating effects could be found regarding the remaining driving-related personality traits and resilience of attention.

### Reaction Speed (RT)

The first control model was significant [*F*(2, 1,875) = 100.33, *p* < 0.001], explaining 10.00% of variance. Only age (β = 0.30, *p* < 0.001) was found to be a significant predictor. Although there is a marginal but significant increase in R^2^ to 10.40% [*F*(4, 1,871) = 4.06, *p* = 0.003] none of the driving-related personality traits included were found to be significant predictors in the model [*F*(6, 1,872) = 36.37, *p* < 0.001]. When including fluid intelligence in the third model there is a significant increase in explained variance to 13.00% [*F*(1, 1,870) = 53.22, *p* < 0.001]. Fluid intelligence (β = −0.17, *p* < 0.001) was found to be a significant predictor in the final model [*F*(7, 1,870) = 39.64, *p* < 0.001]. Therefore, fluid intelligence decreases reaction speed by every point by 2.51, indicating a higher level of fitness to drive.

Regarding mediation analysis via fluid intelligence, a significant total effect of emotional instability on reaction speed could be found (β = 0.06, *p* = 0.01). In detail, the results showed an insignificant direct effect of emotional instability on the cognitive domain (β = 0.02, *p* = 0.44) while the indirect effect through fluid intelligence was found to be significant [β = 0.04, 95% (0.03–0.06)]. Therefore, potential complete mediating effects by fluid intelligence regarding the relation between emotional instability and reaction speed can be noted. However, no further significant mediating effects via fluid intelligence could be analyzed regarding the remaining driving-related personality traits.

### Physical Motor Speed (RT)

In the first step when control variables are included, significant results of the ANOVA [*F*(2, 1,875) = 246.65, *p* < 0.001] can be found, explaining 20.80% of the variance. Age (β = 0.42, *p* < 0.001) and gender (β = 0.14, *p* < 0.001) are significant predictors. When driving-related personality traits are added in the second step a significant increase in R^2^ to 22.00% could be found [*F*(4, 1,871) = 6.97, *p* < 0.001]. Emotional instability (β = 0.08, *p* < 0.001) and self-control (β = −0.06, *p* = 0.034) are significant predictors in the model [*F*(6, 1,871) = 87.91, *p* < 0.001]. While emotional instability is a positive predictor increasing the dependent variable by 2.67 for every point indicating lower fitness to drive, self-control serves as a negative predictor, reducing physical motor speed by 2.20 for every point reflecting a higher level of fitness to drive. When including fluid intelligence in the last model there is again a significant change in explained variance to 24.00% [*F*(1, 1,870) = 43.81, *p* < 0.001]. While emotional instability (*p* = 0.003) and self-control (*p* = 0.04) remained significant, fluid intelligence (β = −0.15, *p* < 0.001) was found to be an additional significant predictor in the final model [*F*(7, 1,870) = 83.33, *p* < 0.001]. Fluid intelligence reduces the dependent variable for every point by 1.45, indicating a higher level of fitness to drive.

When fluid intelligence was included as mediator variable, a significant total effect between emotional instability and physical motor speed could be estimated (β = 0.12, *p* < 0.001). In addition, a significant direct effect of emotional instability was found (β = 0.08, *p* < 0.001), however, also the indirect effect mediated through fluid intelligence showed statistical significance (β = 0.05, *p* < 0.001). Therefore, it can be concluded that the relationship between physical motor speed and emotional instability is partially mediated by fluid intelligence. Again, no additional significant mediating effects could be found regarding the remaining driving-related personality traits and physical motor speed.

### Perceptual Speed (TAVT)

In the first step of the analysis, significant model results can be shown [*F*(2, 1,879) = 358.11, *p* < 0.001] explaining 27.60% of the variance. Age (β = −0.51, *p* < 0.001) and gender (β = −0.07, *p* < 0.001) are significant predictors. When adding driving-related personality traits in the second step a significant increase in explained variance to 30.60% can be found [*F*(4, 1,875) = 19.96, *p* < 0.001]. Additionally, emotional instability (β = −0.16, *p* < 0.001) as well as a sense of responsibility (β = 0.06, *p* = 0.011) are significant predictors in the second model [*F*(6, 1,875) = 137.50, *p* < 0.001]. Emotional instability reduces the perceptual speed by 0.29 for every point, indicating a lower level of fitness to drive, while a sense of responsibility increases the independent variable by 0.09, reflecting a higher level of fitness to drive. When fluid intelligence is included in the last step there is a significant increase in R^2^ to 37.30% [*F*(1, 1,874) = 200.28, *p* < 0.001]. Fluid intelligence (β = 0.28, *p* < 0.001) was found to be a significant predictor in the final model [*F*(7, 1,874) = 158.99, *p* < 0.001]. Therefore, fluid intelligence increases the dependent variable by 0.16 for every point, indicating a higher level of fitness to drive.

Regarding fluid intelligence as a mediator, a significant total effect of emotional instability and perceptual speed could be found (β = −0.20, *p* < 0.001). Furthermore, a significant direct effect of emotional stability on the cognitive domain was estimated (β = −0.13, *p* < 0.001) while also the indirect effect via fluid intelligence showed statistical significance [β = −0.07, 95% (−0.09 to −0.05)]. As a result, it is noted that the relation between emotional stability is potentially partially mediated by fluid intelligence. However, no further significant mediating effects could be found regarding the remaining driving-related personality traits and perceptual speed.

### Short-Term Memory (VISGED)

The first model resulted in significant ANOVA results [*F*(2, 612) = 52.13, *p* < 0.001] explaining 14.60% of variance. Only age (β = −0.38, *p* < 0.001) can be found to be a significant predictor. When driving-related personality traits are included there is a significant increase in R^2^ to 16.70% [*F*(4, 608) = 3.84, *p* = 0.004]. In addition, emotional instability (β = −0.08, *p* = 0.035) is a significant predictor in the second model [*F*(6, 608) = 20.26, *p* < 0.001]. Emotional stability as a negative predictor reduces short-term memory by 0.10 for every point, indicating a lower level of fitness to drive. Finally, when including fluid intelligence, a significant increase in explained variance to 24.70% is shown [*F*(1, 607) = 64.38, *p* < 0.001]. Although emotional instability could not remain significant (*p* = 0.17), fluid intelligence (β = 0.32, *p* < 0.001) is shown to be a significant predictor in the last model [*F*(7, 607) = 28.38, *p* < 0.001]. Therefore, fluid intelligence increases short term memory for every point by 0.12, reflecting a higher level of fitness to drive.

The analysis with fluid intelligence as a mediator showed a significant total effect regarding the relationship between emotional stability and short-term memory (β = −0.19, *p* < 0.001). Additionally, a significant direct effect between emotional stability on the cognitive domain was found (β = −0.13, *p* < 0.001) while also the indirect effect mediated by fluid intelligence showed statistical significance [β = −0.07, 95% (−0.09 to −0.05)]. Possible partial mediation through fluid intelligence on the relation between emotional stability and short-term memory can therefore be stated.

## Discussion

The primary goal of this study was to investigate the predictive value of driving-related personality traits and fluid intelligence on cognitive domains reflecting fitness to drive. In the second part we focused on possible mediating effects regarding compensating for deficits.

The results of our regression analyses regarding sociodemographic variables showed that age in particular has a significant statistical relationship with all of the cognitive functions included here. Therefore, we conclude that higher age is linked to lower performance in the tests regarding hand-eye-coordination, selective attention, resilience of attention, reaction speed, physical motor speed, perceptual speed and short-term memory, reflecting a lower level of fitness to drive. These findings are in accordance with a great body of literature which emphasizes age-related declines in the cognitive domains of memory, attention, executive functions as well as visuospatial skills (e.g., Lezak et al., 2012; Salthouse, 2012; Murman, 2015). In addition, gender was found to be a significant predictor in almost all of the above-mentioned cognitive functions except selective attention and short-term memory. Although significant differences in male and female subjects are given, the interpretation of these results does not lead to valid conclusions due to the over-representation of men in the sample. Gender was therefore included in the regression analyses largely for control purposes.

Regarding driving-related personality traits, when controlled for gender and age, emotional instability seems to be significantly linked to hand-eye coordination quality, selective attention, resilience of attention, physical motor speed, perceptual speed and short-term memory. The results show that participants with higher emotional instability reached lower performance levels in tests regarding the above-mentioned cognitive abilities, indicating a lower level of fitness to drive. Our findings are in agreement with Sommer et al. (2008b), who found out that emotional instability is one of the most important factors when predicting driving performance, linking higher levels to lower levels of fitness to drive. Moreover, our results are supported by recent studies which found significant associations between neuroticism, a construct which emotional instability is based on, and decreased cognitive performances (e.g., Sutin et al., 2019; Biernacki and Lewkowicz, 2020). Additionally, our results are supported by Biernacki and Tarnowski (2011), who found out that the sympathetic nervous system is activated more easily in subjects with higher emotional instability, resulting in a cascade of stress hormones being released and thus impairing cognitive performance (e.g., Wolf et al., 2001). Following this, the chronic experience of stress and the corresponding hormones are also linked to damaging the structure of brain regions, for example the hippocampus, which is responsible for cognitive tasks (e.g., Jackson et al., 2011).

Regarding a sense of responsibility, when controlling for age and gender we found statistically significant relationships to hand-eye coordination quality and perceptual speed. Our results showed that test persons with a higher sense of responsibility achieved greater levels of cognitive performance, indicating a higher level of fitness to drive. These findings are supported, for example, by the studies of Sommer et al. (2008b) as well as Šucha and Černochová (2016), which found similar associations between a greater sense of responsibility and a higher level of fitness to drive. One possible explanation for this relationship is that the concept of driving includes a variety of social norms which are represented by formal rules in legislation (Bierhoff, 1998; Bacher, 2000). In order to interact harmoniously with other drivers in traffic, strictly following these social norms is important since deviations are often accompanied by a high risk of negative outcomes (e.g., car accidents, fines, etc.), a greater sense of responsibility may therefore be linked to a higher level of fitness to drive (Bacher, 2000).

When controlling for age and gender, self-control was found to be a significant predictor regarding the outcome physical motor speed. The results showed that greater self-control was associated with higher performances reflecting a higher level of fitness to drive. Our findings are in accordance with the studies of Colom et al. (2007) and Rammstedt et al. (2018), who noted similar effects. It is theorized that a low level of self-control is associated with more rash actions without thinking about long-term consequences, which lead to more accidents in a variety of situations (O’Gorman and Baxter, 2002). Hence, lower self-control resulting in a higher degree of impulsiveness in traffic functions as a predictor of fitness to drive, which is also supported by the study by Šucha and Černochová (2016).

Finally, fluid intelligence, when controlled for age and gender, was significantly associated with all of the cognitive domains included. Our results show that participants with higher performance levels in fluid intelligence also had higher test results in hand-eye coordination speed and quality, selective attention, resilience of attention, reaction speed, physical motor speed, perceptual speed and short-term memory, thus indicating a higher level of fitness to drive. Our findings are supported by a great body of literature stating equal associations between fluid intelligence and all cognitive domains (e.g., Sommer et al., 2008b; Hartshorne and Germine, 2015; Hird et al., 2016; Cochrane et al., 2019).

Mediation analyses showed some interesting compensation effects regarding driving-related personality traits. The relationship between emotional instability and hand-eye-coordination, selective attention as well as reaction speed were fully mediated by fluid intelligence. Since a low level of emotional instability is associated with high fluid intelligence, we conclude that the negative direct effects of emotional instability on hand-eye-coordination quality, selective attention and reaction speed potentially vanish in the context of fluid intelligence. In contrast we found partially mediated effects regarding the relation between emotional instability and resilience of attention, physical motor speed, perceptual speed and short-term memory. Therefore, we conclude that the negative direct effects of emotional instability on the remaining cognitive abilities do not vanish completely but are potentially buffered by higher fluid intelligence and are henceforth slightly reduced.

In general, some limitations have to be mentioned regarding our results. Firstly, our sample consisted of 87.8% male participants resulting in an underrepresentation of women. Although we controlled for gender in our regression analyses, valid separated statistics for gender were problematic. Therefore, we highly recommend that further studies acquire a balanced sample of male and female subjects. Secondly, future research may focus on certain age groups when researching this topic. Since higher age was significantly associated with lower performance in all cognitive domains, it would seem to be prudent to test certain different age groups in order to assess crucial discrepancies in age. Therefore, our results are limited regarding their interpretation for different age groups as well as for detailed information about gender differences. Adding to this, since we used out a cross-sectional design, we recommend that future research should make use of longitudinal studies in order to assess valid temporal exposure changes between driving-related personality traits, fluid intelligence and cognitive abilities in the context of the fitness to drive.

We must mention that for the psychological tests of cognitive abilities in our study, faking bad is extremely unlikely because the participants need good results to regain their driving licenses. Faking good in the performance tests is not possible in this setup. Faking in the sense of giving socially desired answers in the IVPE driving-related personality questionnaire is likely for a certain number of participants. The questionnaire has had an honesty scale for a certain amount of time, but it was removed by the provider over the years, so no conclusive information is available here in detail. Efforts were made to develop non-fakeable driving-related personality tests for traffic psychology (Viswesveran and Ones, 1999; Torner, 2008; Arendasy et al., 2011), but no significant progress has been made in developing better personal inventories, so the IVPE (and its similar successor, the IVPE-R) questionnaire, is still state of the art in Austria.

Our findings provide an interesting starting point for further research regarding the relationship between driving-related personality, cognitive abilities, fluid intelligence, age and driving behavior. The results showed that fluid intelligence had a significant impact on all of the cognitive variables. Emotional instability was found to be have significant relationships with almost every cognitive domain, while a sense of responsibility and self-control were only partially associated. Finally, we found compensating effects of fluid intelligence regarding emotional instability in almost all cognitive functions. Rehabilitation programs for impaired drivers and prevention programs should incorporate the knowledge provided by our article. Traffic psychologists can incorporate our findings when assessing opportunities to compensate for deficits.

## Data Availability Statement

The datasets generated for this study are available on request to the corresponding author.

## Ethics Statement

The studies involving human participants were reviewed and approved by Austrian Academy of Psychology (AAP). The patients/participants provided their written informed consent to participate in this study.

## Author Contributions

MN had the project lead, initiated the research, and provided the data set. BS and TV contributed significantly to the research design and was the methods and statistics lead. AN and AB suggested improvement and gave various comments.

## Conflict of Interest

The authors declare that the research was conducted in the absence of any commercial or financial relationships that could be construed as a potential conflict of interest.
